# Supplementary figures and images for: Effect of vascular resection for perihilar cholangiocarcinoma: a systematic review and meta-analysis
Source: PeerJ. 2021 Sep 23;9:e12184. doi: 10.7717/peerj.12184 (PMC8466000; doi:10.7717/peerj.12184)

A

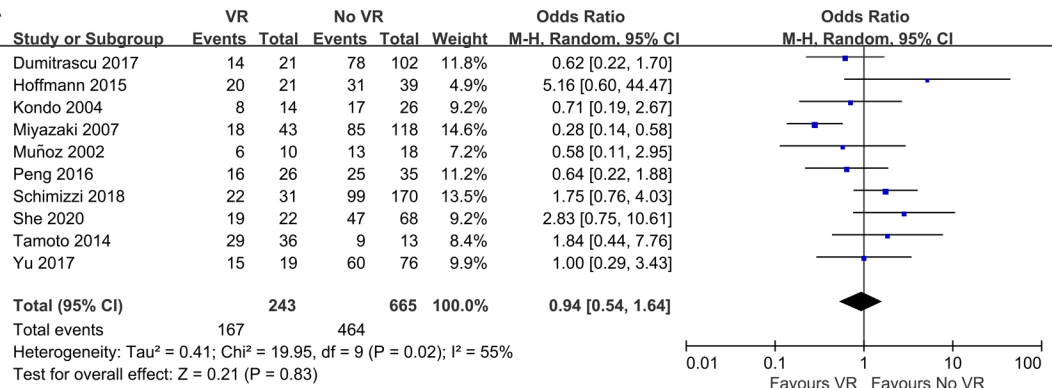

B

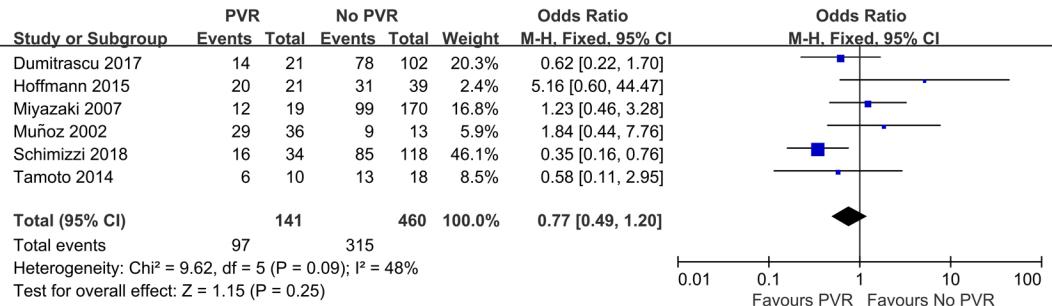

C

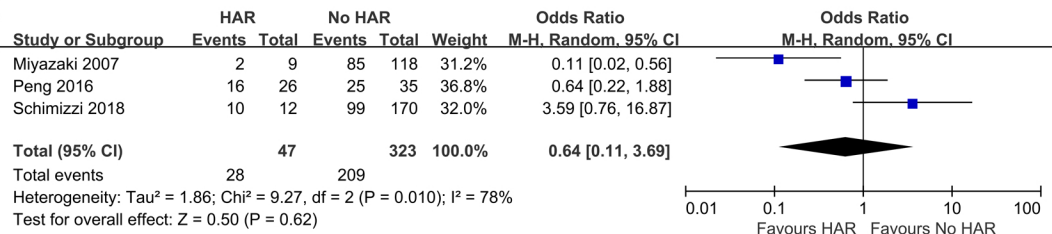

Supplement: Supplemental Information 1 — (A) 1-year OS in patients with and without VR; (B) 1-year OS in patients with and without PVR; (C) 1-year OS in patients with and without HAR. [file peerj-09-12184-s001.pdf]

**A**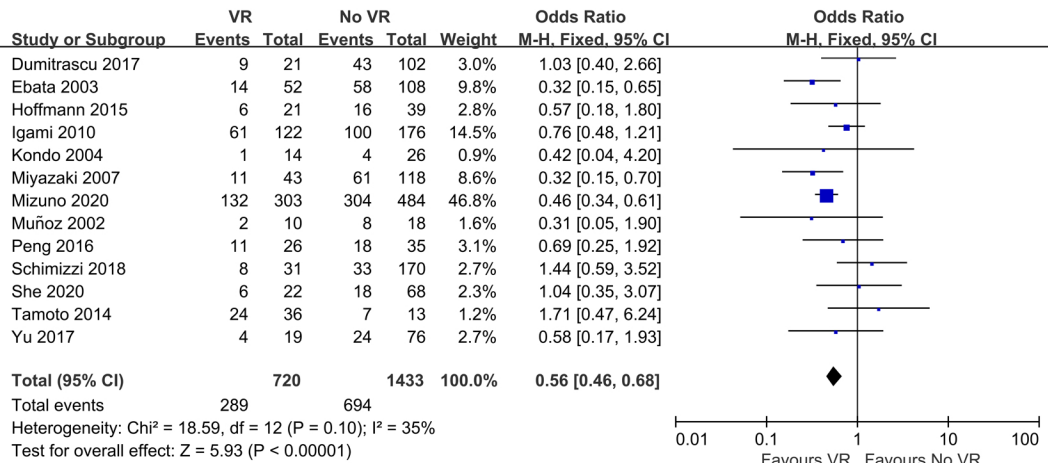**B**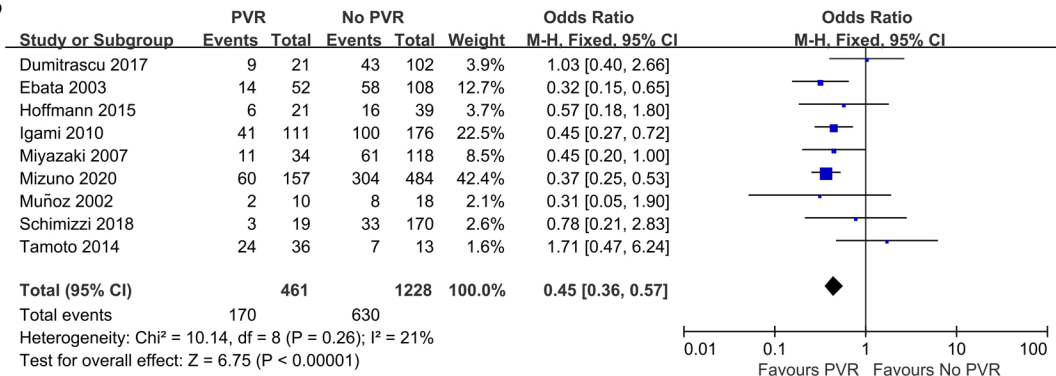**C**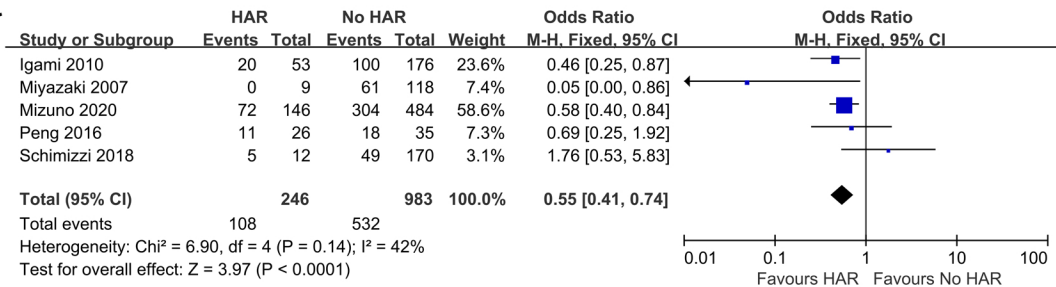

Supplement: Supplemental Information 2 — (A) 3-year OS in patients with and without VR; (B) 3-year OS in patients with and without PVR; (C) 3-year OS in patients with and without HAR. [file peerj-09-12184-s002.pdf]

**A**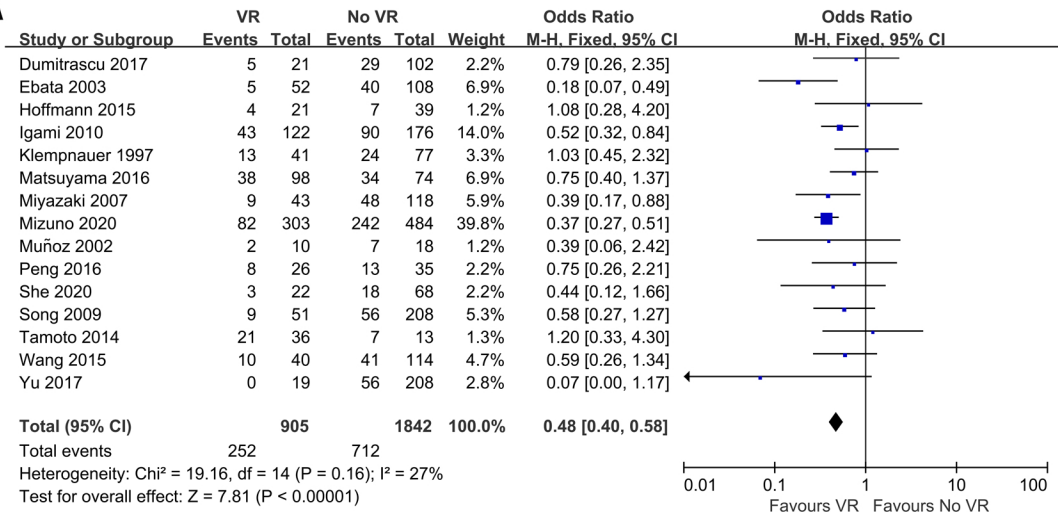**B**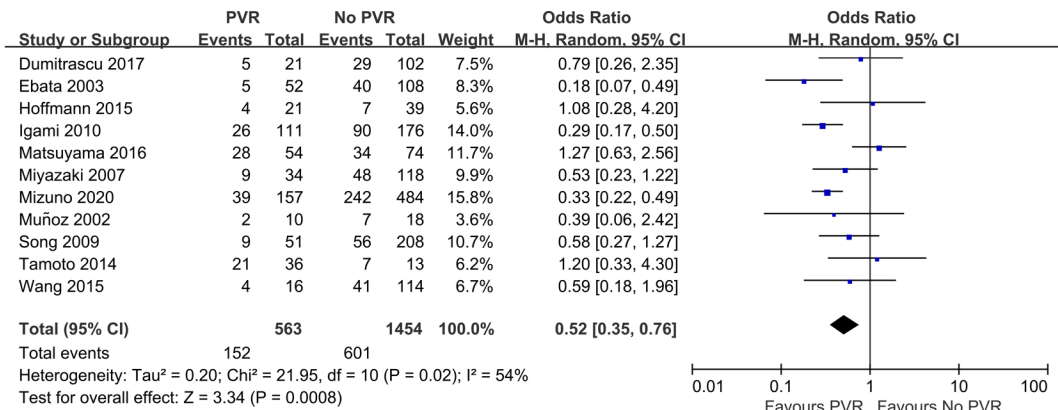**C**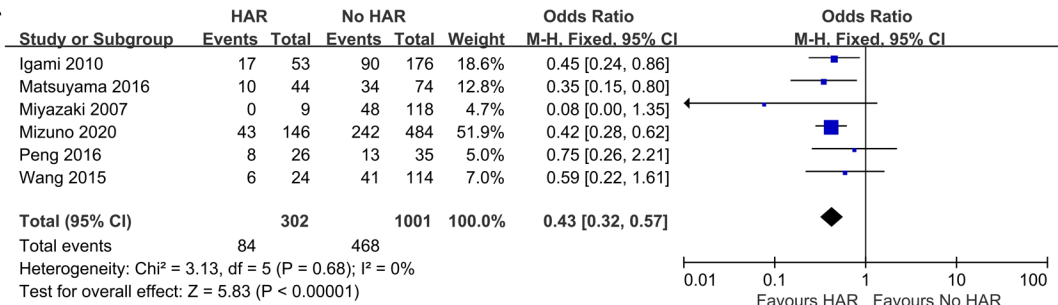

Supplement: Supplemental Information 3 — (A) 5-year OS in patients with and without VR; (B) 5-year OS in patients with and without PVR; (C) 5-year OS in patients with and without HAR. [file peerj-09-12184-s003.pdf]

A

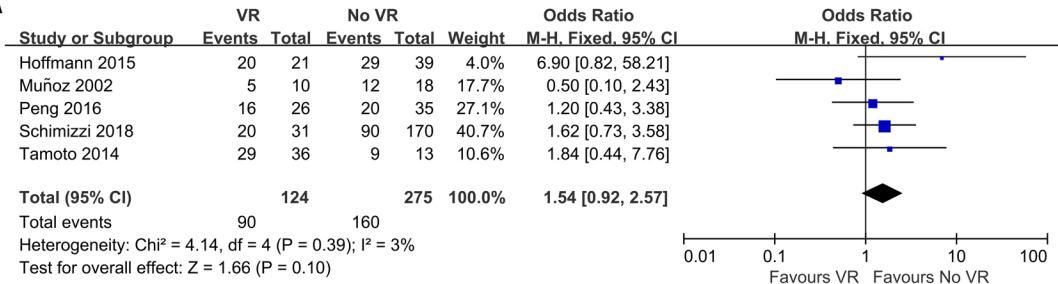

B

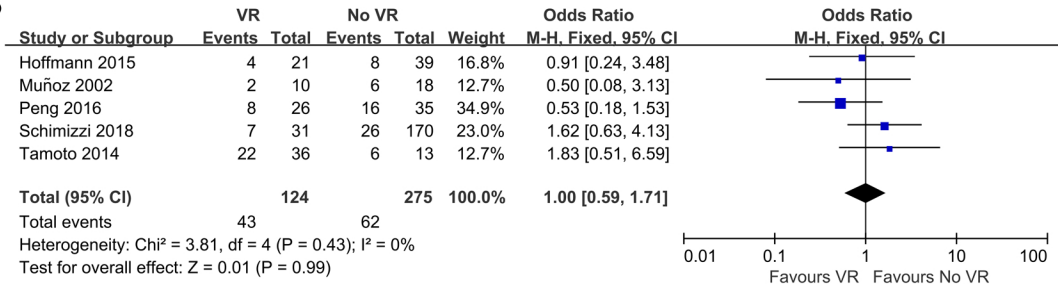

C

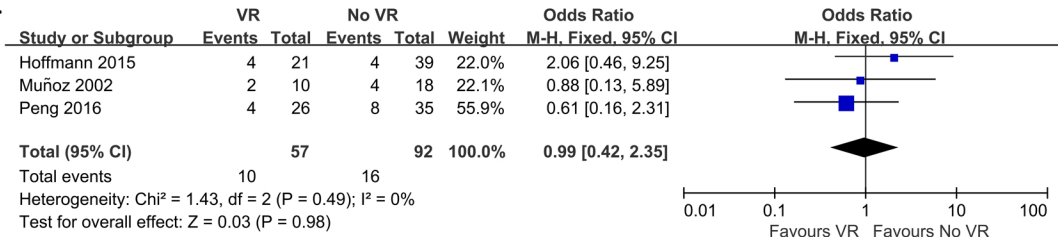

Supplement: Supplemental Information 4 — (A) 1-year DFS; (B) 3-year DFS; (C) 5-year DFS. [file peerj-09-12184-s004.pdf]

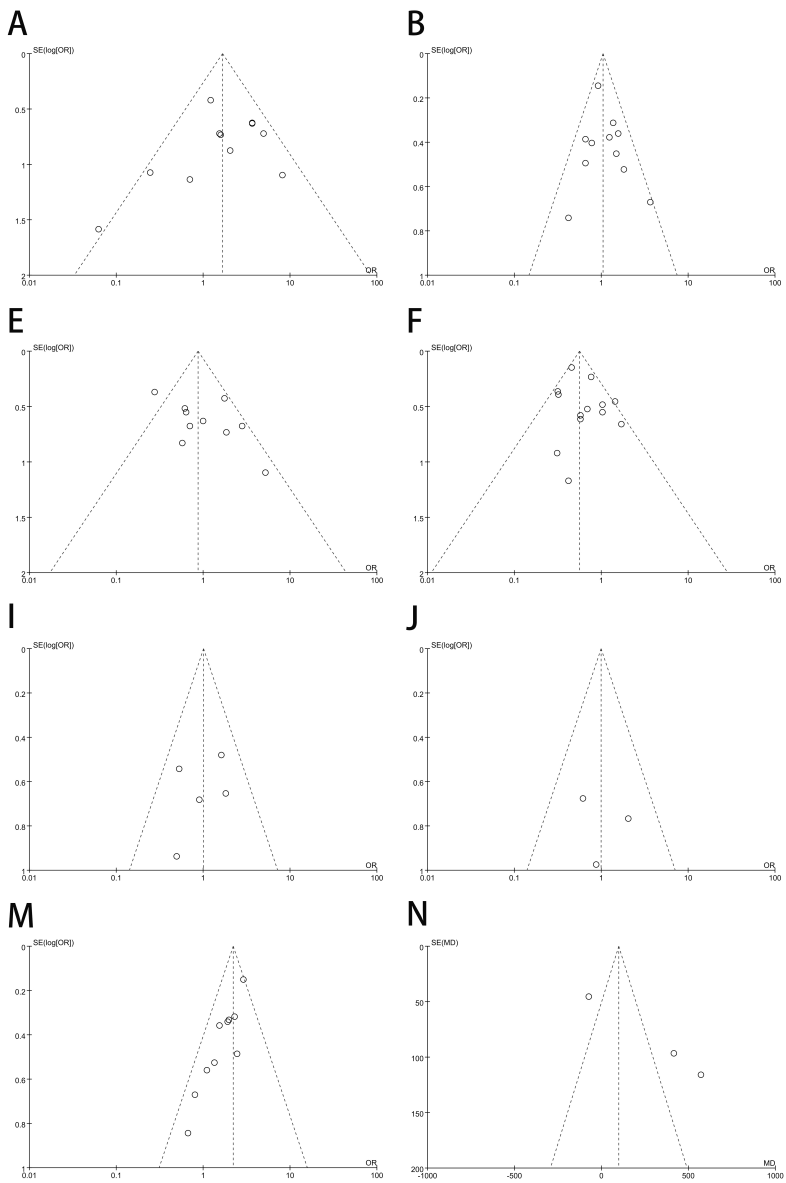

Supplement: Supplemental Information 5 — (A) 90-day mortality rate; (B) overall mortality rate; (C) PHI rate; (D) R0 resection rate; (E) 1-year OS; (F) 3-year OS; (G) 5-year OS; (H) 1-year DFS; (I) 3-year DFS; (J) 5-year DFS; (K) proportion of Ⅲ、Ⅳ stage according to UICC staging systems; (L) positive vascular invasion rate; (M) mean lymph node metastasis rate; (N) intraoperative blood loss . [file peerj-09-12184-s005.pdf]

**A**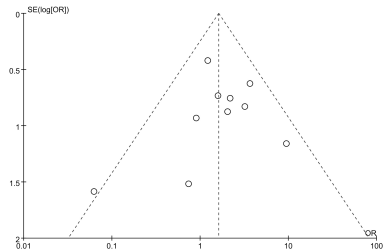**B**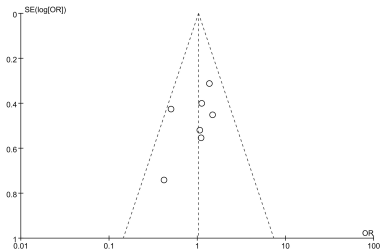**C**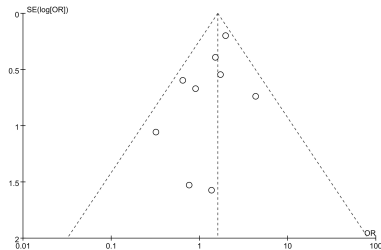**D**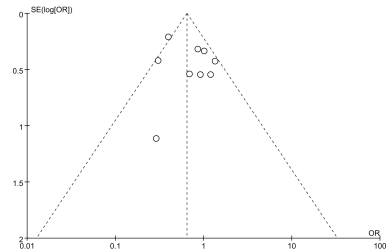**E**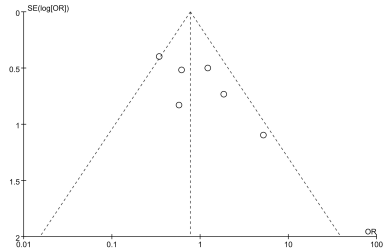**F**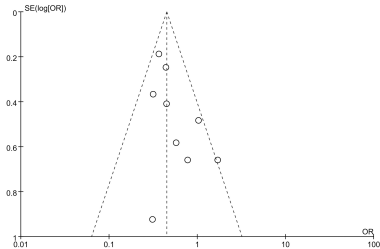**G**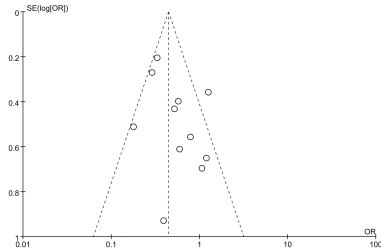**H**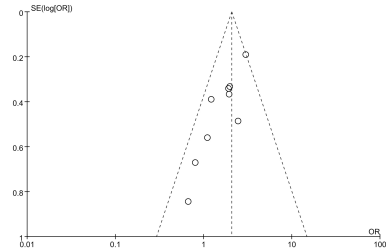

Supplement: Supplemental Information 6 — (A) 90-day mortality rate; (B) overall mortality rate; (C) PHI rate; (D) R0 resection rate; (E) 1-year OS; (F) 3-year OS; (G) 5-year OS; (H) mean lymph node metastasis rate. [file peerj-09-12184-s006.pdf]

**A**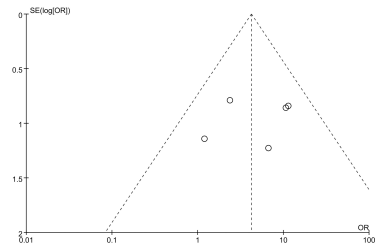**B**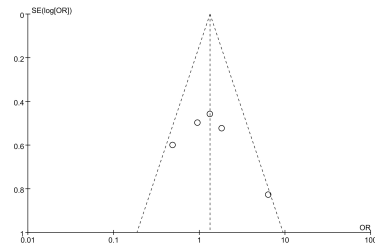**C**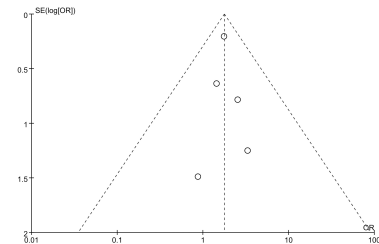**D**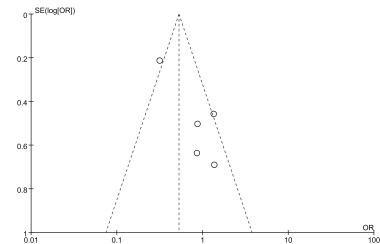**E**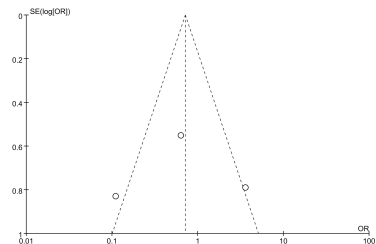**F**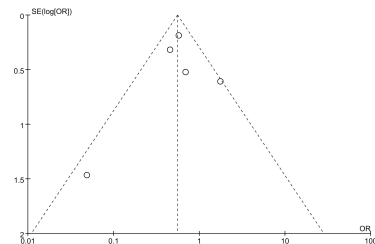**G**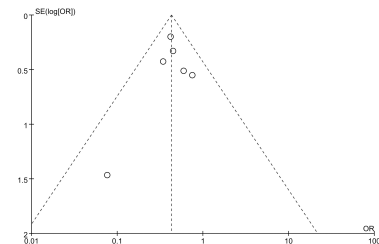**H**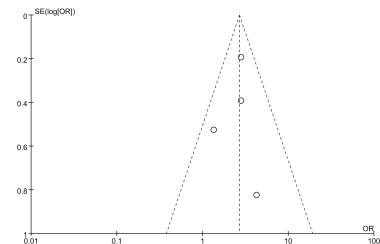

Supplement: Supplemental Information 7 — (A) 90-day mortality rate; (B) overall mortality rate; (C) PHI rate; (D) R0 resection rate; (E) 1-year OS; (F) 3-year OS; (G) 5-year OS; (H) mean lymph node metastasis rate. [file peerj-09-12184-s007.pdf]
