# Supplementary material for: Effect of vascular resection for perihilar cholangiocarcinoma: a systematic review and meta-analysis
Source: PeerJ. 2021 Sep 23;9:e12184. doi: 10.7717/peerj.12184 (PMC8466000; doi:10.7717/peerj.12184)
Supplement: Supplemental Information 9 [file peerj-09-12184-s009.docx]

Table S1 Quality of the included studies assessed by the Newcastle-Ottawa scale (NOS)

| **Study** | **Representativenessof the exposed**  **cohort** | **Selection of**  **the non**  **exposed**  **cohort** | **Ascertainment**  **of exposure** | **Outcome**  **not present**  **at start** | **Comparability**  **of cohorts** | **Assessment**  **of outcome** | **Long enough**  **follow up (≥2**  **years)** | **Adequacy of**  **follow up of cohorts** | **Quality**  **score** |
| --- | --- | --- | --- | --- | --- | --- | --- | --- | --- |
| Wang 2015 | 1 | 1 | 1 | 1 | 1 | 1 | 0 | 1 | 7 |
| Dumitrascu 2017 | 1 | 1 | 1 | 1 | 1 | 1 | 1 | 1 | 8 |
| Ebata 2003 | 1 | 1 | 1 | 1 | 1 | 1 | 0 | 1 | 7 |
| Nagino 2010 | 1 | 1 | 1 | 1 | 1 | 1 | 1 | 1 | 8 |
| Hoffmann 2015 | 1 | 1 | 1 | 1 | 1 | 1 | 0 | 1 | 7 |
| Peng 2016 | 0 | 1 | 1 | 1 | 1 | 1 | 0 | 1 | 6 |
| Schimizzi 2018 | 1 | 1 | 1 | 1 | 0 | 1 | 0 | 1 | 6 |
| Hemming 2011 | 1 | 1 | 1 | 1 | 1 | 1 | 1 | 1 | 8 |
| Tamoto 2014 | 0 | 1 | 1 | 1 | 1 | 1 | 0 | 1 | 6 |
| Higuchi 2019 | 1 | 1 | 1 | 1 | 1 | 1 | 0 | 1 | 7 |
| Lee 2010 | 1 | 1 | 1 | 1 | 1 | 1 | 0 | 1 | 7 |
| Igami 2010 | 1 | 1 | 1 | 1 | 1 | 1 | 0 | 1 | 7 |
| She 2020 | 1 | 1 | 1 | 1 | 1 | 1 | 0 | 1 | 7 |
| Kondo 2004 | 1 | 1 | 1 | 1 | 1 | 1 | 0 | 1 | 7 |
| Jong 2012 | 1 | 1 | 1 | 1 | 1 | 1 | 0 | 1 | 7 |
| Miyazaki 2007 | 1 | 1 | 1 | 1 | 1 | 1 | 0 | 1 | 7 |
| Muñoz 2002 | 1 | 1 | 1 | 1 | 1 | 1 | 1 | 1 | 8 |
| Klempnauer 1997 | 1 | 1 | 1 | 1 | 1 | 1 | 0 | 1 | 7 |
| Matsuyama 2016 | 1 | 1 | 1 | 1 | 1 | 1 | 1 | 1 | 8 |
| Yu 2017 | 1 | 1 | 1 | 1 | 1 | 1 | 1 | 1 | 8 |
| Mizuno 2020 | 1 | 1 | 1 | 1 | 1 | 1 | 1 | 1 | 8 |
| Song 2009 | 1 | 1 | 1 | 1 | 1 | 1 | 0 | 1 | 7 |
