# Supplementary material for: Effect of vascular resection for perihilar cholangiocarcinoma: a systematic review and meta-analysis
Source: PeerJ. 2021 Sep 23;9:e12184. doi: 10.7717/peerj.12184 (PMC8466000; doi:10.7717/peerj.12184)
Supplement: Supplemental Information 10 [file peerj-09-12184-s010.docx]

Table S2 Search strategy

| **Database** | **Search keywords** |
| --- | --- |
| **Pubmed** | 1 “Klatskin’s tumour” [all]  2 “hilar bile duct carcinoma” [all] OR “hilar bile duct cancer” [all] OR “hilar cholangiocarcinoma” [all] OR “hilar bile duct neoplasm” [all] OR “hilar bile duct tumour” [all] OR “perihilar cholangiocarcinoma” [all]  3 (“surg*” [all] OR “resect*” [all] OR “segmentect*” [all] OR “hepatect*” [all]) AND (“liver” [all] OR “hepatic” [all])  4 “hepatectomy” [MeSH Terms]  5 #1 OR #2  6 #3 OR #4  7 #5 AND #6  8 “vascular resection” [all] OR “vascular reconstruction” [all] OR “angiectomy” [all]  9 “hepatic artery resection” [all] OR “portal vein resection” [all]  10 #8 OR #9  11 #7 AND #10 |
| **Embase** | 1 ‘Klatskin tumor’ OR ‘hilar bile duct carcinoma’  2 ‘hilar bile duct cancer’ OR ‘hilar cholangiocarcinoma’ OR ‘hilar bile duct neoplasm’ OR ‘hilar bile duct tumour’ OR ‘perihilar cholangiocarcinoma’  3 ‘liver resection’  4. ‘(‘liver’ OR ‘hepatic’) AND (‘surgery’ OR ‘resection’ OR ‘segmentectomy’ OR ‘hepatectomy’)  5 #1 OR #2  6 #3 OR #4  7 #5 AND #6  8. ‘vascular resection’ OR ‘vascular reconstruction’ OR ‘angiectomy’  9 ‘hepatic artery resection’ OR ‘portal vein resection’  10 #8 OR #9  11 #7 AND #10 |
| **COCHRANE** | 1 Klatskin’s tumour  2 (hilar bile duct carcinoma) OR (hilar bile duct cancer) OR (hilar cholangiocarcinoma) OR (hilar bile duct neoplasm) OR (hilar bile duct tumour) OR (perihilar cholangiocarcinoma)  3 ((surg*) OR (resect*) OR (segmentect*) OR (hepatect*)) AND ((liver) OR (hepatic))  4 hepatectomy  5 #1 OR #2  6 #3 OR #4  7 #5 AND #6  8 (vascular resection) OR (vascular reconstruction) OR (angiectomy)  9 (hepatic artery resection) OR (portal vein resection)  10 #8 OR #9  11 #7 AND #10 |
