# Supplementary material for: Effect of vascular resection for perihilar cholangiocarcinoma: a systematic review and meta-analysis
Source: PeerJ. 2021 Sep 23;9:e12184. doi: 10.7717/peerj.12184 (PMC8466000; doi:10.7717/peerj.12184)
Supplement: Supplemental Information 12 [file peerj-09-12184-s012.docx]

**The rationale for conducting the systematic review / meta-analysis.**

Cholangiocarcinoma is a rare adenocarcinoma that originates from the epithelial cells of bile ducts. Perihilar cholangiocarcinoma (PHC) is the main type of cholangiocarcinoma, accounting for 50% to 67% of cases. The prognosis of PHC is generally poor because of its anatomical location and aggressive biology. Resection surgery and transplantation are the main treatment methods for PHC that provide a chance of long-term survival. The median overall survival (OS) of patients with PHC who undergo curative resection varies from 19 to 39 months.

The objective of surgery is to achieve R0 resection. However, PHC usually adheres to or is surrounded by vessels, such as the portal vein or hepatic artery, which makes curative resection difficult to achieve. Therefore, to achieve R0 resection, vascular resection (VR) can be performed during the operation. It has been reported that the proportion of VR during PHC surgery ranges from 15% to 38%. VR refers to portal vein resection (PVR), hepatic artery resection (HAR) or both. Although VR is performed in many circumstances, controversy still exists. For PVR, portal vein involvement by PHC was previously considered a sign of unresectability. With the development of surgical techniques, PVR has been performed at several clinical centers. However, the efficacy and safety of PVR for PHC are controversial. Ebata et al. reported that combined portal vein and liver resection can offer long-term survival to some selected patients with advanced PHC. However, Hoffmann et al. found that PVR greatly increased the perioperative morbidity rate and had no benefit for PHC in terms of the oncologic outcomes. In addition, surgical resection with simultaneous HAR for PHC is a demanding procedure. Similar to PVR, attitudes toward HAR remain inconsistent. Miyazaki et al. reported that HAR had no beneficial effect on prognosis and led to an increase in the perioperative morbidity and mortality rates; thus, the use of HAR may not be justified. Nagino et al. demonstrated that major hepatectomy with HAR could offer a better chance of long-term survival in selected PHC patients. Given these conflicting recommendations, the efficacy and safety of PVR and HAR for treating PHC patients need to be further clarified.

**The contribution that it makes to knowledge in light of previously published related reports, including other meta-analyses and systematic reviews.**

To date, several meta-analyses have been performed to evaluate the efficacy and safety of VR for PHC patients; however, the results of these studies were inconsistent. By including 2457 patients, Abbas et al. found that PVR may result in survival benefits for some patients with advanced PHC, which was similar to Chen’s study. However, Wu et al. and Yu et al. found that PVR increases postoperative mortality and morbidity and worsens long-term survival; thus, surgical decisions should be made cautiously. For HAR, Abbas et al. and Yu et al. found that HAR is associated with increased mortality and morbidity without proven survival benefits for PHC patients. In a recent guideline for cholangiocarcinoma from Italy, PVR was recommended when there was unilateral portal vein invasion. However, the recommendation for PVR in this study was limited with a low quality of evidence due to the small number of related studies. Further, hardly any attention was given to HAR in the Italian study. Therefore, we critically appraised the existing literature in an updated systematic review and meta-analysis, which included several newly published articles that were not included in previous meta-analyses. Besides a large number of studied populations, we also distinguish between PV and HA resection. And the results showed PVR did not increase the postoperative morbidity rate and slightly increase the postoperative mortality rate. HAR did not increase the postoperative morbidity rate and significantly increased the postoperative mortality rate. Neither PVR nor HAR improved R0 resection rate or long-term survival. Consequently, PVR is relatively safe and might confer benefits to certain patients with advanced PHC in terms of long-term survival. HAR is related to increased mortality and has not been demonstrated to benefit long-term survival, which should be considered before performing this procedure. This study systematically reviews and statistically evaluates the effect of VR, including PVR and HAR on short - and long-term outcomes in PHC patients. This meta-analysis will provide some reference significance for clinicians to choose the appropriate surgical plan for PHC patients.
